# Dataset of some added-lignin thermoformed pulps

**DOI:** 10.1016/j.dib.2024.111176

**Published:** 2024-11-27

**Authors:** Jost Ruwoldt

**Affiliations:** RISE PFI AS, Høgskoleringen 6B, 7491 Trondheim, Norway

**Keywords:** Added-lignin thermoformed pulps, Lignin, Kraft lignin, Soda lignin, Thermoforming, Molded pulp, CTMP, NBSK

## Abstract

This data article summarizes the material properties of some added-lignin thermoformed pulps (ALTPs). This type of molded pulp is particularly suited for replacing plastics in environments, where moisture is encountered, as the lignin reduces the transport and adsorption of water. The dataset was measured on wet formed substrates with either softwood chemi-thermomechanical pulp (CTMP) or northern bleached softwood Kraft pulp (NBSK). To the basis weight of 500 g/m^2^ pulp fibers (6 g pulp per 15 cm by 8 cm substrate area) were added 0.8 g of lignin, constituting a loading of 13.33 % added lignin per dry fibers. Right after forming, the sheets were pressed at 20 bar and room temperature between blotting paper to remove excess water, air dried overnight, and subsequently thermopressed at 175 °C and 200 bar. The resulting materials were measured and weighed, cut into test stripes with 15 mm width, and tensile tested. The absorption of water (water uptake) was measured after immersing the test pieces (15 mm by 15 mm) in distilled water for 24 h. The data shows slight increases in density after lignin addition and, on average, a reduction in water absorption. In particular, adding acetylated lignin significantly lowered the water uptake, as compared to the regular soda or Kraft lignin. The tensile strength and stiffness of CTMP was the same or lower after lignin addition, whereas NBSK showed increases in both stiffness and strength.

Specifications Table

**Table:** 

Subject	*Material Science: Polymers and Plastics*
Specific subject area	*Material data on lignocellulose fiber composites (mechanical and chemical pulp) with added lignin biopolymer as binder and property modulator*
Type of data	*Table*
Data collection	*Wood pulp-lignin composites were first made by wet-forming, drying, and subsequent pressing at elevated temperature and pressure. Specimen of these composites were cut out, measured, and weighed to determine the basis weight and density. Five specimen measuring 15mm by 15mm were immersed in water for 24h to measure the water-uptake. Specimen stripes (70mm by 15mm) were tensile tested in a universal material tester by Zwick-Rhoel, Germany.*
Data source location	*RISE PFI AS, Høgskoleringen 6B, 7491 Trondheim, Norway*
Data accessibility	Repository name: Zenodo Data identification number: 10.5281/zenodo.11203446 Direct URL to data: 10.5281/zenodo.11203446 The repository file is an .xlsx/type file accessible with Microsoft Excel. On “Sheet1” in the file, the first line indicates the type of information (description of sample composition or values) stated in the same column (rows 3 to 12), while the second line indicates the unit of the corresponding values. Each row between row 3 and 12 delineates the measured, processed, and averaged values relating to the sample composition given in the first and second column. For each data point, both the average and standard deviation are given in subsequent cells. On “Sheet2” of the same file, the raw data of tensile testing (strain versus tensile force) is given. Here, row 1 column 1 and 2 are descriptors indicating the pulp fibers and added lignin type of each specimen and measurement in the row 1 and 2, columns 2 to 121. Row 3 lists the type of measured value (strain or standard force), to which the corresponding value unit is given in row 4. Rows 5 and below in column 2 to 121 list the measured values, were each column with strain values is followed by the column giving the force values to the corresponding measurement.
Related research article	*None*

## 1 Value of the Data

• This data may serve as a guide for packaging engineers that are using molded pulp materials.
• It provides new insights into biobased binders for cellulose fiber materials.
• The data may benefit paper engineers and scientists in developing new materials.
• It helps to advance research on added-lignin thermoformed pulps.
• It may hence promote the development of sustainable substitutes for fossil-based plastics.

## 2 Background

Molded pulp is usually encountered as three-dimensional packaging materials, which have been made from wood pulp. Traditionally, a pulp dispersion is filtrated into the mold, which is followed by casting and convection drying or in-mold drying. In one implementation, the material is additionally pressed in the mold during drying, which has received considerable attention in the scientific community lately [1,2]. These materials are also referred to as thermoformed pulp products, which can be produced by either wet- or dry-forming processes [3]. While posing as a sustainable alternative for plastics packaging, these materials still exhibit some of the traditional challenges encountered in cellulosic materials, such as proneness to moisture [4]. The addition of lignin has therefore been proposed recently, producing so called added-lignin thermoformed pulps (ALTPs), which exhibit reduced wetting and less absorption of water [5]. It has furthermore been shown that the lignin acts as a binder, confining fiber-swelling and reducing the overall proneness to wetting [6]. Despite the potential of these new materials, little research has been done to evaluate possible formulations. This data article hence presents some data on ALTPs with varying pulp, lignin type, and chemical modification by acetylation.

## 3 Data Description

**Fig. 1:**
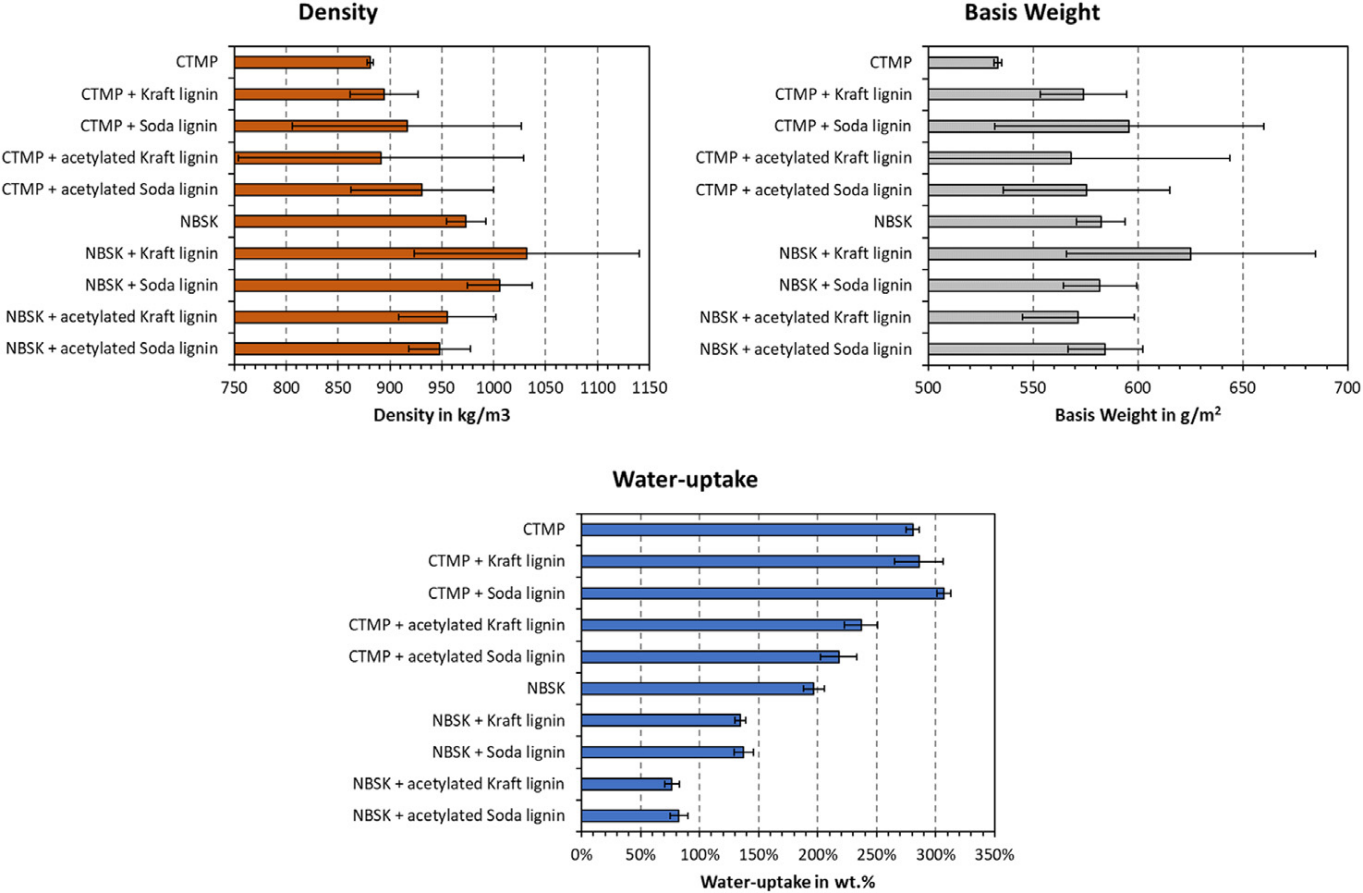
Density, basis weight, and water-uptake of the tested ALTPs. Data on blank CTMP and NBSK was taken from a previous publication [7].

The density, basis weight, and water-uptake of the different samples is plotted in Fig. 1. These values are also found on “Sheet1” of the data repository. As can be seen, the basis weight of ALTPs was closely within 567 g/m^2^, which corresponds to 13.33 % added lignin per dry fiber of pulp. Minor increases in density were noted for the CTMP composites, which are in line with previous results [5,6]. Still, the data scattering is significant, in addition to NBSK noting a decrease in density after addition of acetylated lignin. The water-uptake was significantly reduced after lignin addition for all tested compositions, except for CTMP with added Kraft or soda lignin. Addition of acetylated lignin always produced a reduction of water-uptake. The decrease in water absorption is greater for NBSK as compared to CTMP.

**Fig. 2:**
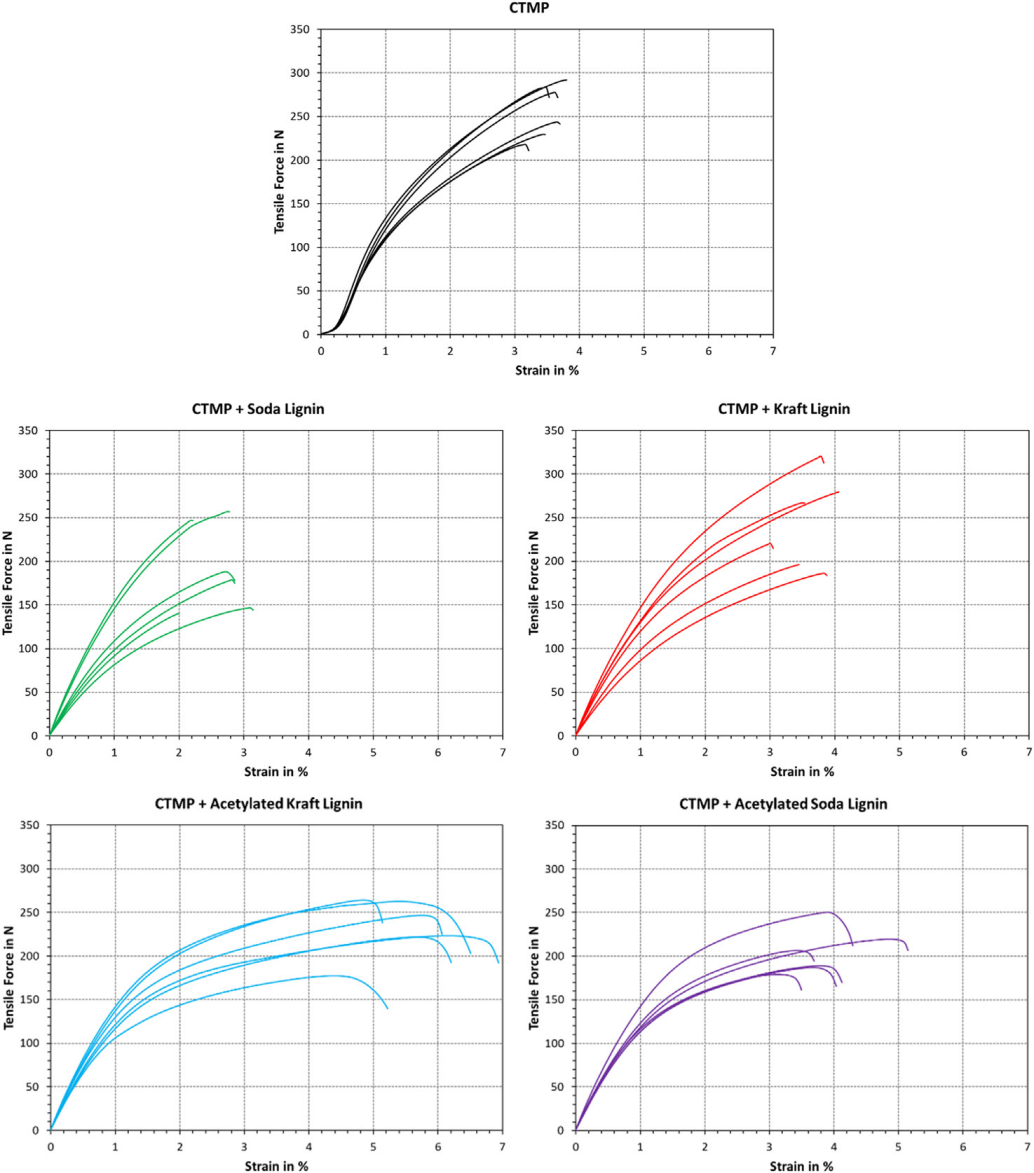
Stress–strain diagrams of ALTPs with CTMP. Data on the blank CTMP sample was reproduced from a previous publication [7].

The stress-strain curves of CTMP and CTMP-based ALTPs are plotted in Fig. 2. The corresponding values are also found on “Sheet2” of the data repository. The curves differ in their qualitative progression, where the blank CTMP sample yielded a lower response of tensile force at the initial elongation (<0.3 %). Compared to this, the addition of Kraft or soda lignin led to a higher slope at values close to the origin. Addition of acetylated lignin shared this initial response; however, the force changes slope, reaching a plateau at elongations above 2 %.

**Fig. 3:**
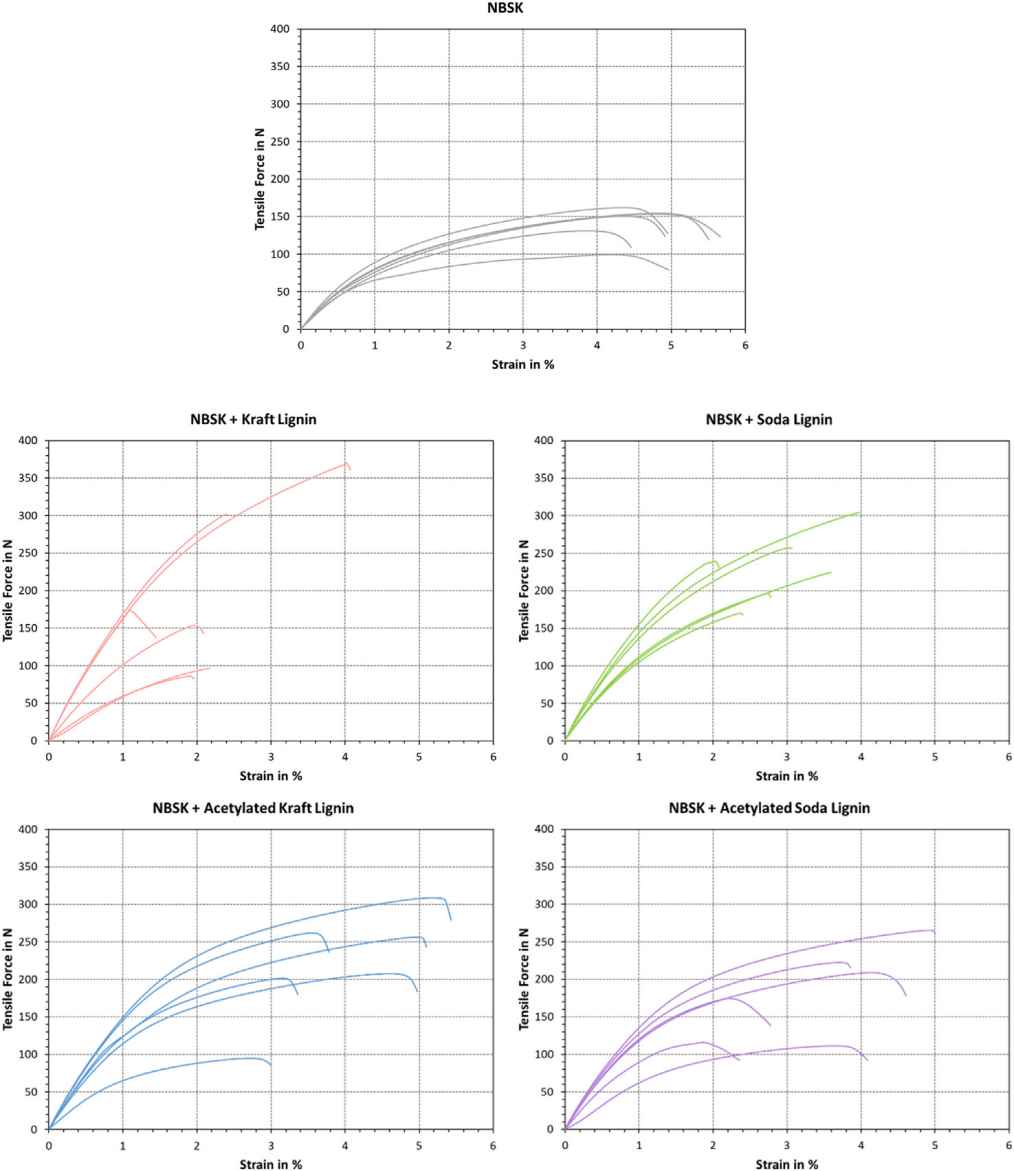
Stress–strain diagrams of ALTPs with NBSK. Data on the blank NBSK sample was reproduced from a previous publication [7].

The stress-strain curves of NBSK and corresponding ALTPs are plotted in Fig. 3. According values are also found on “Sheet2” of the data repository. Here, the pristine pulp showed a similar progression of CTMP-based ALTPs with acetylated lignin, i.e., an initially higher slope that led to a plateau at elongations greater than 2 %. Addition of acetylated Kraft or soda lignin mirrored this progression qualitatively, albeit at a higher force and lower elongation at break. Addition of untreated Kraft or soda lignin did not yield the same plateau at higher elongations and a significantly lower elongation at break.

**Fig. 4:**
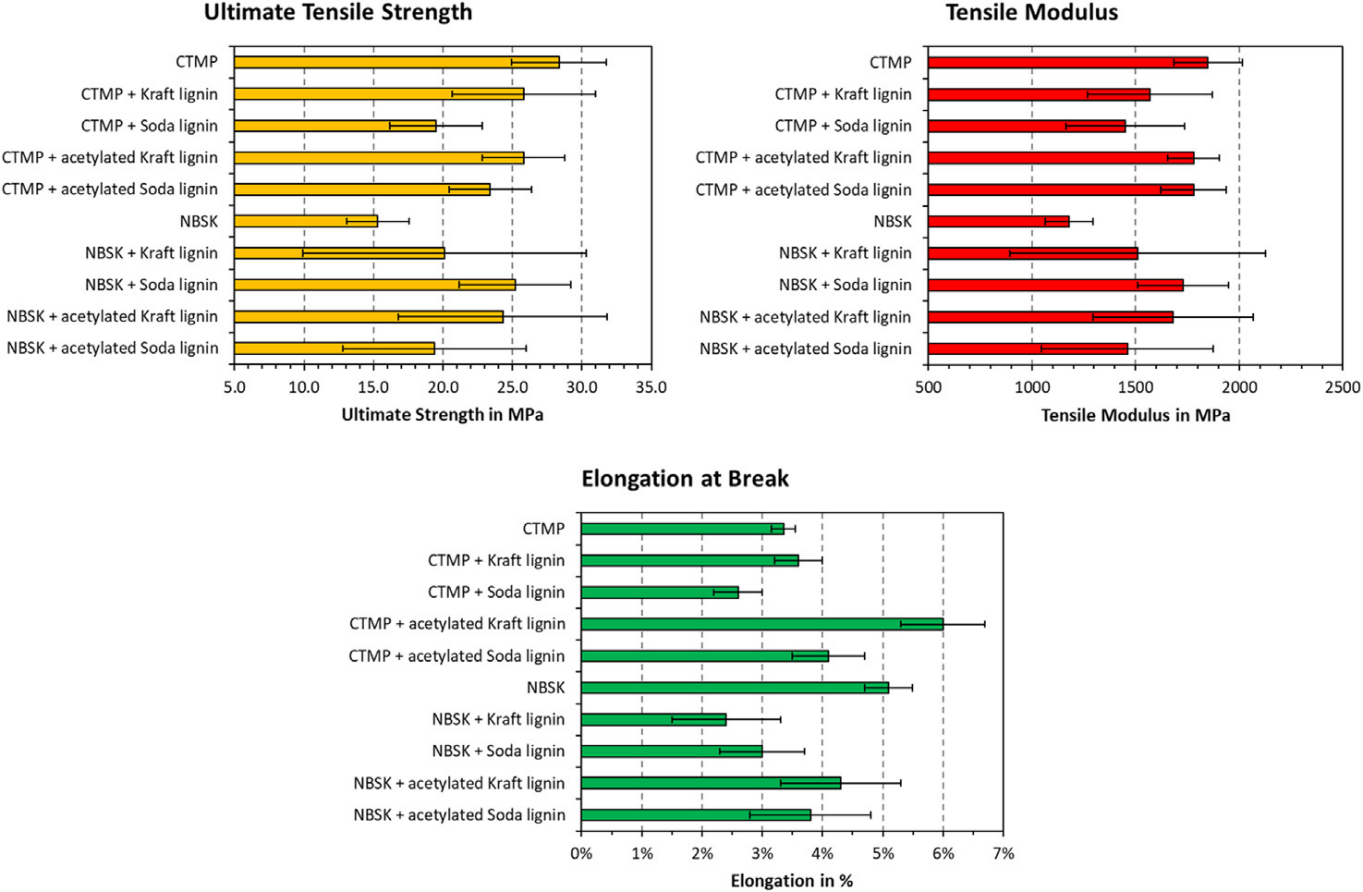
Ultimate tensile strength, stiffness modulus, and elongation at break of the tested ALTPs. Data on blank CTMP and NBSK was taken from a previous publication [7].

The summary of obtained tensile strength, stiffness, and elongation at break is plotted in Fig. 4. The corresponding values are also found on “Sheet1” of the data repository. In general, the results mirror the observations made from the stress-strain diagrams in Fig. 2, Fig. 3. Overall, the addition of lignin produced a reduction in ultimate tensile strength for CTMP, while the strength of NBSK was increased. The reduction of tensile modulus in CTMP was less pronounced, whereas NSBK also showed significant increases in stiffness after lignin addition. The elongation at break increased for CTMP with added acetylated lignin. NSBK responded also with a reduction in elongation at break after lignin addition, which was more pronounced for the unmodified lignin samples.

## 4 Experimental Design, Materials and Methods

Bleached softwood chemi-thermomechanical pulp (CTMP) with a Canadian standard freeness (CSF) of 450 and an ISO brightness of 80 was provided by FollaCell AS, Norway. Northern bleached softwood Kraft (NBSK) of proprietary origin was provided by MoRe Research AB, Sweden. The two pulps were characterized in detail in a previous study [7]. Kraft lignin (BioPiva 395) was supplied by UPM Biochemicals, Finland. Soda lignin (Protobind 2000) was purchased from PLT Innovations, Switzerland. A cationic flocculant by the name PCB 20 was provided by Solenis Norway AS, Norway. Reagents for acetylation were obtained as pyridine (≥99.7 %, analytical reagent for KarlFischer titration) and acetic anhydride (≥99 %, ReagentPlus) from VWR (Norway) and Merck Sigma-Aldrich (Norway), respectively. Deionized water was used in this study.

For acetylation, the lignin was first dried at 55 °C for 5 h under vacuum. To 1 g of lignin, 10 ml of pyridine were then added, and the lignin dissolved. Another 10 ml of acetic anhydride were then added, the sample vial closed and stored in an exicator for 2 d The acetylated lignin was then retrieved by quenching the reaction in ice-cold water, filtration, and washing of the filter-cake with 200 ml water. The filter-cake was then laid out to dry at ambient conditions, yielding the acetylated lignin.

The experimental methods for preparing the thermoformed pulp are based on the procedures published in previous articles [5,7]. The pulp was first disintegrated according to ISO 5263 using a Lorentzen & Wettre disintegrator at 30 000 revolutions and 15 g/l consistency in hot water (> 80 °C) and subsequently diluted to 5 g/l. This pulp suspension was stored for at least 24 h prior to further handling. Handsheets were formed by gravity filtration onto a metallic mesh with an area of 15 cm by 8 cm, targeting a weight of 6 g dry fibers, i.e., a basis weight of 500 g/m^2^. Before filtration, 0.8 g lignin were added together with 200 ppm cationic flocculant per dry fiber weight. Afterwards, the wet handsheets were retrieved and pressed between blotting paper at 20 bar and ambient temperature. The handsheets were further laid out to dry at ambient conditions, yielding a moisture content of approximately 10 wt%. The dried handsheets were then thermopressed at 200 bar and 175 °C for 5 min. Each handsheet composition was made an analyzed in duplicates.

Prior to analysis, the thermopressed handsheets were equilibrated at 50 % relative humidity and 23 °C for at least 24 h, as stated in ISO 187. Fibers extending over the 15 cm by 8 cm surface area were cut off and the thickness was measured at five predetermined positions according to ISO 534. The density was calculated by dividing the weight by the surface area times the averaged thickness. Stripes measuring 70 mm by 15 mm were cut and tensile tested in a Zwick-Rhoel material tester using a load cell of 2.5 kN at an elongation speed of 100 mm/min. The distance between the clamps was 40 mm. Six stripes were tested per sample composition. The water-uptake was measured by cutting out five pieces of 15 mm by 15 mm area, submersing these in water for 24 h, and measuring the weight before and after. The water uptake *p_water_* was calculated according to Eq. (1), using the weight of the sample equilibrated at 50 % relative humidity and 23 °C *m*_50 %_*_RH_* and the weight of the sample immersed in water for 24 h *m_water_*.

(1) $$p_{water}=\frac{m_{water}-m_{50\%RH}}{m_{50\%RH}}$$

## Limitations

No obstacles were encountered during the sample preparation and analysis, which would hamper interpretation of the data in this article.

## Ethics Statement

This data article does not involve any human or animal subjects and has no data collected from social platforms.

## Credit Author Statement

**Jost Ruwoldt:** Conceptualization, methodology, validation, formal analysis, ressoruces, data curation, writing – original draft, writing – review & editing, visualization, supervision, project management.

## Data Availability

ZenodoDataset of some added-lignin thermoformed pulps (Original data).
